# Prevalence of Tuberculosis and HIV co infection with respect to CD4 counts in Chengalpattu Medical College and Hospital

**DOI:** 10.1186/1471-2334-14-S3-P24

**Published:** 2014-05-27

**Authors:** M Malathi, A Vijayalakshmi, CP Ramani, P Balapriya, R Kulothungan

**Affiliations:** 1 Department of Microbiology, Chengalpattu Medical College, Chengalpattu, India; 2 ART Center, Chengalpattu Medical College, Chengalpattu, India

## Background

Infection with HIV increases the risk of tuberculosis. HIV and TB co infection is associated with increased number of mortality. This study aimed to characterize the patients of HIV with co infection of TB and their CD4 counts.

## Methods

This study is a retrospective study by assessment of the patient`s medical records. Study period is from January 2013 to November 2013. 575 patients with CD4 count less than 500 were selected as the study population. Among them, 37 patients were identified with HIV and TB co infection. Their clinical presentations were also studied.

## Results

The prevalence of HIV and TB co infection is 6.43%. Co infection is more prevalent in men (67.56%) with a mean age of 42.12years. Co infection in female is 27.02% with a mean age of 40.4 years. Pediatric co infection rate is 5.40%. Pulmonary tuberculosis (70.27%) is the most frequent form of TB followed by tuberculous lymphadenitis (29.72%).

## Conclusion

From this retrospective study, it has been confirmed that the prevalence of HIV and TB co infection is more common in patients with CD4 count less than 500. This study reflects the co-ordinate active participation of RNTCP and Integrated Counseling and Testing centre (ICTC) in identifying the HIV patients who are at risk of developing co infection.

